# A systematic review of asymptomatic *Plasmodium knowlesi* infection: an emerging challenge involving an emerging infectious disease

**DOI:** 10.1186/s12936-022-04339-8

**Published:** 2022-12-06

**Authors:** Nurul Athirah Naserrudin, Mohd Rohaizat Hassan, Mohammad Saffree Jeffree, Richard Culleton, Rozita Hod, Kamruddin Ahmed

**Affiliations:** 1 Department of Community Health, Faculty of Medicine, Universiti Kebangsaan Malaysia, Kuala Lumpur, Malaysia; 2 Borneo Medical and Health Research Centre, Faculty of Medicine and Health Sciences, Universiti Malaysia, Sabah, Kota Kinabalu, Malaysia; 3 Sabah State Health Department, Ministry of Health, Kota Kinabalu, Malaysia; 4 Department of Community and Family Medicine, Faculty of Medicine and Health Sciences, Universiti Malaysia Sabah, Kota Kinabalu, Malaysia; 5 Division of Molecular Parasitology, Proteo-Science Center, Ehime University, Toon, Japan; 6 Department of Pathology and Microbiology, Faculty of Medicine and Health Sciences, Universiti Malaysia Sabah, Kota Kinabalu, Malaysia

**Keywords:** *Plasmodium knowlesi*, Zoonotic malaria, Asymptomatic infection, Asymptomatic characteristic, Malaria epidemiology

## Abstract

**Background:**

In the last decade *Plasmodium knowlesi* has been detected in humans throughout South East Asia. The highest risk groups for this infection are males, adults and those performing forest-related work. Furthermore, asymptomatic cases of *P. knowlesi* malaria have been reported including among women and children.

**Methods:**

Pubmed, Scopus and the Web of Science databases for literature describing asymptomatic *P. knowlesi* malaria published between 2010 and 2020 were searched. A systematic literature review was conducted to identify studies reporting the prevalence and incidence of laboratory confirmed asymptomatic *P. knowlesi* cases in humans, their clinical and demographic characteristics, and methods used to diagnose these cases.

**Results:**

By analysing over 102 papers, thirteen were eligible for this review. Asymptomatic *P. knowlesi* infections have been detected in 0.03%–4.0% of the population depending on region, and infections have been described in children as young as 2 years old. Various different diagnostic methods were used to detect *P. knowlesi* cases and there were differing definitions of asymptomatic cases in these studies. The literature indicates that regionally-differing immune-related mechanisms may play a part on the prevalence of asymptomatic *P. knowlesi*.

**Conclusion:**

Differing epidemiological characteristics of asymptomatic *P. knowlesi* malaria in different regions reinforces the need to further investigate disease transmission mechanics. Effective public health responses to changes in *P. knowlesi* epidemiology require proactive intervention and multisectoral collaboration.

**Supplementary Information:**

The online version contains supplementary material available at 10.1186/s12936-022-04339-8.

## Background

*Plasmodium knowlesi* is an emerging public health challenge in South East Asia [1].The incidence of *P. knowlesi* malaria is highest in Malaysian Borneo where the first large focus was found in Sarawak, Malaysia [2]. The infection mainly occurs in forested areas where monkeys and humans coexist [3, 4]. Genetic characterization has identified three subpopulations of *P. knowlesi;* those of Peninsular Malaysia and Borneo, with the latter population further subdivided depending on the macaque host species (*Macaca fascicularis* or *Macaca nemestrina*) subpopulations [5].

The majority of *P. knowlesi* malaria infections cause low parasitaemia and mild clinical manifestations [6]. Likewise, in Sabah, Malaysia the case fatality rate (CFR) was 1.70/1000 [7]. Although it may reach high parasitaemia and can lead to lethal infections in humans [7]. The high risk groups are female, age ≥ 45 years, and patients with comorbidities (e.g. including pregnancy), requires early diagnosis and management to avoid fatalities [7]. Although most mild cases are treatable successfully with chloroquine [7], its effectiveness for severe cases requires further investigation [7]. An estimated 6 to 9% of cases are severe; a rate similar to that caused by *Plasmodium falciparum* [8]. Severe *P. knowlesi* malaria can deteriorate rapidly and become fatal if treatment is delayed, a factor linked to its 24 h hour replication cycle in the blood. The commonly misdiagnosed *P. knowlesi* as other species of malaria (e.g. *P. falciparum*) added to the need of rapid, sensitive and specific diagnosis in endemic areas [6]. The availability of intervention, anti-malarials and recommendation to administer/rapid access to IV artesunate at an early stage of the infection can mitigate severity and relieve chronic infection [6, 7].

In the last decade, asymptomatic *P. knowlesi* infections have been increasingly frequently detected in humans [9, 10]. This may be due to improved molecular based diagnostics that can detect sub-microscopic infections, and by a increasing awareness of the disease [11, 12]. Asymptomatic *P. knowlesi* malaria is, therefore, an emerging public health concern compounded by ambiguities caused by different diagnostic methods and infection outcomes [10, 11]. The reasons for the increasing number of human cases of *P. knowlesi* malaria remain largely unknown, however, evidences suggest that anthropogenic activities (e.g. land-use changes) may be partly responsible [13]. A systematic review highlighted the role of human behaviour that put the vulnerable communities at risk [14]. There is still no scientific consensus, for example, on whether the disease is entirely zoonotic, or whether human to human transmission can occur [15].

Individuals with submicroscopic infections can infect mosquitoes, and may contribute to malaria transmission [16]. There is concern, therefore, that submicroscopic asymptomatic *P. knowlesi* malaria may constitute an infectious reservoir for disease transmission. The discrepancy between the incidence and the reported seroprevalence gives a sense of the extent of asymptomatic cases in the population [17]. The introduction of a specific control program for zoonotic malaria should be prioritized to mitigate this situation [18]. Therefore, it is critical to design an effective *P. knowlesi* malaria surveillance and control programme.

Here, literature review on *P. knowlesi* was performed to determine the prevalence, the epidemiological characteristics, and the diagnostic criteria used for asymptomatic *P. knowlesi* malaria. This is the first systematic review to describe the burden of asymptomatic *P. knowlesi* malaria infection.

## Methods

Articles were searched from three databases [Scopus, Web of Science (WoS) and PubMed] and gray literatures. The CoCoPop framework was used to address the aim of the review relevant to prevalence or incidence [19]. ‘Condition’ refers to the variable of interest: Asymptomatic *P. knowlesi* infection among humans as host. ‘Context’ refers to information regarding how the condition will be measured, diagnosed, or confirmed. ‘Population’ refers to the study population or study subjects [19]. Articles published from January 2000 through December 2020 were searched. The search terms were ("asymptomatic") AND ("*knowlesi*" OR "*Plasmodium knowlesi*" OR "simian malaria") AND ("world*" OR "community" OR "population") using Boolean operators "OR" or "AND" also truncation.

### Eligibility criteria

The inclusion criteria were any study published in English that reported laboratory confirmed asymptomatic *P. knowlesi* cases. Exclusion criteria were any asymptomatic *P. knowlesi* study with no related context including case reports and case series, editorial, animal studies, drug studies, proceedings, short communications and experimental studies. Further excluded were articles on malaria in pregnancy, co-infection with other infection (besides other *Plasmodium* species), and review articles.

### Data selection and extraction

To reduce the risk of bias, studies were assessed by three authors (NABN, MRH and RH) independently to identify relevant articles based on the title and abstract. All articles were reviewed by NA, MRH, and RH. All three authors, searched references for additional relevant studies. In case of discrepancies, it was resolved by consensus by a third (KA), fourth reviewer (MSJ), and fifth reviewer (RC). The data set of this systematic review, including the name of the author (s), publication year, study design, country of study (distribution), study period, diagnostic method, the prevalence or incidence, sociodemographic factor, other relevant factors, were extracted into Microsoft Excel (Microsoft Corporation, Redmond, Washington, USA), for further analysis.

### Criteria for asymptomatic *P. knowlesi* infection

The criteria for asymptomatic *P. knowlesi* infection were based on the definition by the World Health Organization (WHO), in which asymptomatic parasitaemia is defined as the presence of asexual parasites in the blood without symptoms of illness [20]. However, if a study had its own clear operational definition of asymptomatic *P. knowlesi* infection, it was accepted for this review.

### Quality of included studies

To assess the quality of the observational studies, the Strengthening the Reporting of Observational Studies in Epidemiology (STROBE) checklist was used, and independently assessed by three reviewers. It has the total of 22 items. Each question based on STROBE was categorized as 'yes' (meet criteria), 'no' (did not meet criteria), or 'not applicable.' A 'partly' option was not included to avoid it being used to negate having to choose between yes and no [21]. The quality of the study is sufficient if it reaches a 70% of description of these items. Following the STROBE checklist, a summary of the quality assessment of the included studies, is presented in Additional file 1: Table S1.

## Results

The literature search generated a total of 43 articles, 20 from Scopus, five results from WoS, and 18 from PubMed. They were 59 studies from backward citation searching references identified from the eligible studies. The backward citation was performed to identify potentially relevant papers from the searched articles, that might be missed at the screening process. The WHO website was searched for grey literature. After duplicates were removed, 55 remained. Following the screening of titles and abstracts, 15 studies were retained for more detailed evaluation. Finally, 13 studies were included in this review after full text reviewing. The most common reason for exclusion were experimental studies, other *Plasmodium* species studies, opinion paper and reviews (Additional file 2: Figure S1).

All the included studies are detailed in Additional file 3: Table S2. There was one longitudinal study, 10 cross sectional studies and one case–control study. Two studies were conducted in Sabah, Malaysia [10, 13], where one study was from Sabah and the southern part of the Philippines [22], two studies from Sarawak, Malaysia [23, 24], two from Vietnam [9, 25], one from Surat Thani, Thailand [26], Cambodia [13], Indonesia [27, 28], Myanmar [17], and along the Laos and Vietnam border [29]. Most of the studies were performed in rural areas. For example, the study which was performed in Surat Thani Thailand included 18 villages in four districts [26]. The study from western Cambodia was conducted in 23 villages [12]. In a study carried out in Indonesia, more than 80 localities across 3 regencies were involved, with different geological land conditions; Batubara—a semi forested and plantation area, Langkat—a forested highland area with altitude 105–530 m above sea level, and South Nias, island cluster in the Indian Ocean [28]. The study that was conducted along the Laos and Vietnam border was performed in an area which is densely forested where various minority groups engaged in forest activities as part of their lifestyle like agriculture and hunting. Malaria occurring in this region is often called “*border malaria”* due to the transmission occurring among forest dwellers and ethnic minority groups that traverse the border between two separate political states [29].

In terms of sampling strategy, all studies were conducted using community-based data. The majority were cross-sectional studies and one was a case–control study performed in Sabah [10].

All studies were carried out during 2009–2019. Some of the studies analyzed the effects of seasons on transmission. One cross-sectional survey was performed during the dry season (January) and the rainy season (May) in Thailand [26]. A study in Myanmar was performed during the rainy season [17]. Another study carried out in Sabah (Kudat and Kota Marudu) analysed the effects the El Nino phenomena with widespread droughts and high smoke pollution before and after the survey [13].

In Thailand, Shimizu et al. (2020) defined asymptomatic malaria as those who were positive for *Plasmodium*, but afebrile within the past 48 h [26]. In Cambodia, asymptomatic subjects were defined as those with tympanic temperature below 37.5 °C, but positive for *P. knowlesi* by polymerase chain reaction (PCR) [12], however the study in Laos-Vietnam border did not specifically mention temperature but their asymptomatic subjects (children) had normal body temperature [29]. Otherwise, in a study done in Aceh Besar, Indonesia, asymptomatic cases were defined as those with negative thick and thin blood smears, but positive for pan-*Plasmodium* Loop-Mediated Isothermal Amplification (LAMP) [27]. In another study done in Indonesia, tests were offered to healthy individuals with axillary temperature < 37.5 °C [28]. In all these studies *P. knowlesi* was identified by nested PCR.

### Statistical analysis

Cases were presented as both absolute numbers and as percentages. The majority of the studies used univariate and multivariate logistic regression analyses to identify potential risk factors (Additional file 3: Table S2). However, some studies only reported the number of asymptomatic cases, but no further analysis was done to evaluate the relationship, association or influence with demographic or other contributing factors.

### Incidence of asymptomatic *P. knowlesi*

The earliest report of asymptomatic *P. knowlesi* was in Vietnam was in 2009 [9], followed by another in 2011 [25], and recently in 2018, along the Laos-Vietnam border [29]. The study area is densely forested and hilly, and the majority of its community lifestyle is based on exploitation of forest products, agriculture, farming, and cultivation of crops. In the former study, three of 95 (3.16%) samples were positive for *P. knowlesi* [9]. In the latter study, 28 of 37 (75.68%) people were from the minorities residing in Khanh Phu were positive. Among them, 2 of the 5 and 2 of the 7 positive subjects from Trinh and Kinh ethnicities, had co-infection with another *Plasmodium* spp. The study done in 2018 detected 7 asymptomatic *P. knowlesi* cases from 35 other asymptomatic *Plasmodium* cases after screening 75 households [29].

There were 1.34% (20 cases out of 1495 samples) asymptomatic cases detected in Aceh Besar, Indonesia by rapid active case detection (RACD) [27]. However, in Batubara, Langkat, and South Nias, Indonesia, they were able to detect more submicroscopic and asymptomatic cases [28].

In Matunggong district of Sabah, 0.03% (three out of 10,100 blood samples) and 0.3% (20 positive out of 1147 blood samples) of individuals were detected as asymptomatic *P. knowlesi* cases [10, 13] in two studies where PCR was used for diagnosis. Two cases were mono-infection, but mixed infections with *P. vivax* were also detected [13].

A study done among the people residing in longhouses in Betong, Sarawak, detected seven (0.23%) asymptomatic *P. knowlesi* cases out of 3002 blood samples [23]. Although there was no symptomatic cases in this study however, a high number of cases was detected from the same area during a study done three year prior to the above study [30].

A study in Cambodia reported eight (0.05%) asymptomatic *P. knowlesi* from 14,732 samples taken from 23 villages [12]. The study even detected the presence of asymptomatic *Plasmodium cynomolgi* in 11 samples. All samples were analysed using nested PCR and positive samples were confirmed by nucleotide sequencing. In Surat Thani Thailand, in January 2019, one case was detected among 7034 subjects, and in May 2019, two cases among 8671 subjects. In both months, cases were analysed by nested PCR using capillary blood samples collected by finger pricking in EDTA tubes [26] rather than directly onto filter paper. In these studies, under-reporting may have occurred as it is thought that this method of blood collection may miss up to 50% of malaria infections [31]. Thus, the true prevalence is likely to be higher than the reported cases.

### Immunity

Asymptomatic *P. knowlesi* infected patients are assumed to have a degree of naturally acquired immunity against the parasite, presumably through previous exposure [10, 22, 28]. It remains unclear whether infection with human malaria species such as *P. falciparum* and *Plasmodium vivax* might confer cross-reacting immunity against *P. knowlesi*. There is an understanding gap of many of the factors surrounding the involvement of immunity in asymptomatic *P. knowlesi* [13].

### Seroprevalence

A study from Sabah, Malaysian Borneo, and Palawan, the Philippines, used enzyme-linked immunosorbent assay to detect antibodies against *P. falciparum, P. vivax* and *P. knowlesi* antigens among the population residing in rural areas [22]. The study population had 7.1% (178/2503) seropositivity to *P. knowlesi* antigen, with the highest prevalence in Limbuak, Pulau Banggi (11.7%; 93/795) followed by Matunggong (6.8%; 79/1162) and Bacungan (1.1%; 6/546) [22].

### Diagnostic methods

Not all studies used microscopy for confirmation of malaria. In these cases, either PCR [9, 10, 12, 13, 17, 22, 24, 26, 28, 29] or LAMP [27] was used to confirm malaria. In studies where thick and thin smears were read, microscopy was performed in addition to PCR. Furthermore, only one study, performed in Cambodia, reported the geometric mean parasite density of patients with asymptomatic *P. knowlesi* infection. The mean geometric parasite density was 52,488 parasites/ml [12].

Moreover, two studies from Indonesia used a RACD policy for close contacts of confirmed *P. knowlesi* patients, which involved microscopic diagnostic screening of all household members of an index case and the neighbours living close to the index case [27, 28].

The type of samples used in these studies were either whole blood collected in EDTA containing tubes [13, 26, 28], or dried blood spot on filter paper [17, 23–25, 27–29]. All samples underwent PCR to detect the presence of *Plasmodium* DNA. The majority of studies used nested PCR to amplify *P. knowlesi* specific 18S ribosomal DNA. Imwong et al*.* used whole genome amplification to characterize the *Plasmodium* species in samples with insufficient DNA volumes [12]. In addition, genes other than 18S ribosomal RNA can be used [12]. The majority of the studies used nested PCR to confirm *P. knowlesi* infection [10, 13, 24, 29].

Shimizu et al. used quantitative PCR (qPCR) able to detect down to 10 copies of the target gene per reaction. All positive samples were tested by nested PCR to determine *Plasmodium* species [26]. LAMP was used to detect *P. knowlesi* cases in Aceh Besar, Indonesia [27], and a study in Myanmar involved whole genome amplification [12].

### Sociodemographic, socioeconomic and environmental conditions

In the majority of studies, questionnaires were used to assess the demographic, socioeconomic and environmental characteristics of *P. knowlesi* cases. The most significant characteristic of asymptomatic *P. knowlesi* malaria is that it predominantly affected males [10, 12, 16, 17, 22, 26]. Studies performed in Sabah, Malaysia also revealed similar results [10, 13]. A male-biased risk was also detected in the Greater Mekong Subregion [29]. However, based on a seroprevalence study, both genders showed similar prevalence of exposure to malaria [22], and in Vietnam men and women were found to be equally infected [25].

Interestingly, asymptomatic children as young as two were found to be infected with *P. knowlesi* by PCR, but not by microscopy [9, 29]. In Vietnam, untreated children with asymptomatic infections were positive for malaria even after one year [9]. However, it is unclear whether these were new infections or the continuation of the same infection. At the Laos-Vietnam border all asymptomatic *P. knowlesi* cases were in children between 2 and 15 years old [29]. In Cambodia, the median age of positive cases were 33.5 years (ranged 23–58) [16], and in Betong, Sarawak only adults were detected with asymptomatic malaria [23]. These findings are in contrast with a study done among the ethnic minorities in Vietnam where the mean age was 16.9 years old [25]. However, in Indonesia, all age groups were affected by asymptomatic *P. knowlesi* infection, and 51.18% (130/254) of the cases were among those above 15 years old [28]. These findings were supported by a study from Myanmar, where all ages group were affected by asymptomatic malaria, and more cases were found in the 5 to 15 year old age group [17]. Again these findings are also supported by a seroprevalence study conducted in Sabah, Malaysia where all age groups were found to have antibodies against *P. knowlesi* [13]. In Surat Thani, Thailand, no specific age group was at higher risk *P. knowlesi* than any other [26].

Those who work in the plantation sector and farmers are at particular risk of asymptomatic *P. knowlesi* infection [13, 22, 23, 27] and have been shown to be seropositive for *P. knowlesi* [13]. In Belaga, Sarawak, farming and forest-based activities such as hunting, fishing and gathering forest products were associated with infection. Other ethnic communities in Sarawak such as the Kenyah community perform agricultural activities such as planting in hilly paddies [24]. Some cases were found among households who did not venture to forest [9, 10, 13, 22, 27]. Having contacts with macaques, occupational related factors such as farming, working in the plantation sector, and socioeconomic status were associated with *P. knowlesi* exposure based on model analysis [13].

Individuals residing within 1 km of the forest, and those living within 500 m of cleared areas have increased risk of *P. knowlesi* infection [13]. Exposure was exclusively related to forest fragmentation due to agricultural expansion and land changes [9, 13, 22, 28]. As a result of ecological changes, there is a possibility of increased contacts with monkeys, which further increases the risk of infection [9, 13, 22, 28].

### Personal vector control measures

A study from Surat Thani, Thailand demonstrated that the use of bed nets and indoor residual spray (IRS) for four months acts as a preventive method against malaria infection. People who practiced self-protection, such as repellent usage and wearing protective clothing were protected from mosquito bites [26]. These results were supported by previous studies from Kudat and Kota Marudu, Sabah where the usage of bed nets and other malaria prevention methods were negatively associated with *P. knowlesi* exposure [13].

In ecological studies, individuals residing in houses less than one meter from ground level and individuals residing at higher geographical elevations had lower risks of *P. knowlesi* exposure [13].

## Discussion

Despite clear anti-malaria guidelines and widespread control efforts, the prevalence of asymptomatic *P. knowlesi* is widespread and worrying. Although currently there are no report of human-mosquito-human transmission, the possibility remains that this occurs. The *P. knowlesi* Evidence Review Group has stated that the exponential rise in *P. knowlesi* cases warrants further genetic, laboratory, and entomological studies on the transmission dynamics of zoonotic malaria [11]. Cross-sectional study design has limitation as it is unable to provide evidence of either new or chronic infection among the study subjects, while longitudinal study could provide some answers related to whether the cases were asymptomatic infections, evidence for possible human to human transmission or clustering of the cases by using molecular detection methods [23]. As asymptomatic cases can last several months, longitudinal study design is more superior to cross-sectional studies to identify these cases. In addition, asymptomatic cases can be pre-symptomatic [26], and the communities exposed to *P. knowlesi* malaria must perceive the threat of the infection, and seek medical treatment appropriately [18]. Asymptomatic *P. knowlesi* cases were detected among all age groups and both genders, including children, women, and those not related to forest related occupations. A major risk factor is habitation proximity to forested areas. Currently limited number of studies addressing asymptomatic *P. knowlesi* malaria and, therefore, more studies are required.

Asymptomatic malaria cases are rarely detected by light microscopy due to relatively low levels of parasitaemia, leading to an underestimation of the prevalence of these types of infections [10, 12, 13, 17, 27]. Furthermore, similarities in morphology between *P. knowlesi*, *P. falciparum* and *Plasmodium malariae* at certain stages of their life cycle may contribute to misdiagnosis during case detection. To avoid this, molecular diagnostic methods are commonly utilized, and these can lead to improved detection of asymptomatic infections [12]. The *P. knowlesi-*specific schizont-infected cell agglutination variant antigens (SICAvar) PCR assay used by Lubis et al*.* identified an unexpectedly large number of *P. knowlesi* infections compared to a similar study from Acheh, Indonesia [28]. The detection of mixed infections of *P. knowlesi* with other *Plasmodium* species, for example *P. vivax* demonstrates the need for specific assays for *P. knowlesi* to overcome possible cross-amplification of (for example) *P. vivax*, which can occur with some 18 s RNA based assays. In addition, *SICAvar* based PCR is suggested to be more specific and sensitive compared with PCRs that target the *cytochrome B* gene [28]. The SICAvar gene assay was specific as it generated bands from the DNA of *P. knowlesi* and not from the other *Plasmodium* species tested in the study (*Plasmodium inui, P. cynomolgi, Plasmodium coatneyi,* and *Plasmodium fieldi, P. falciparum, P. vivax, P. malariae, Plasmodium ovale curtisi*, *P. ovale wallikeri*). The assay is sensitive as it was estimated, to detect as low as 0.1 parasite per µL of whole blood [28]. The robustness of using this method was further confirmed by PCR amplification and sequencing where it exhibited high variability of the conserved exon and able to detect distinct sequences of *P. knowlesi* reference genome [28]. LAMP has also been used to detect *P. knowlesi* infections [12]. Next-generation sequencing also offers a promising approach [32].

The underestimation of asymptomatic cases skews epidemiological parameters such as incidence rate, and this can lead to suboptimal guideline design. Thus, sub-optimal diagnostic methodology can hamper efforts to determine disease incidence and are detrimental to public health.

Although males and adults are usually at higher risk of *P. knowlesi* malaria due to work-related exposure, the infection also affects women and children, indicating that a further understanding of the risk factors for exposure is required. In Vietnam, children were found to harbour the parasite a year after an initial positive result. As the children did not receive treatment after the initial screening, it is possible that these infections were chronic and persistent, although re-infection cannot be ruled out. Future studies could incorporate genotyping assays to differentiate between infections [9].

Several gaps remain in the understanding of asymptomatic *P. knowlesi* infection. Particularly, it is unclear why *P. knowlesi* infection in certain cases remains asymptomatic while others develop clinical symptoms. Furthermore, immunity related factors have been described to play a role in contributing to the *P. knowlesi* malaria disease outcome [22, 26]. In addition, inadequately treated primary infection can contribute to low level parasitaemia [16]. Further research should be carried out at a more comprehensive population range in *P. knowlesi* endemic regions, focusing on the inflammatory and immune responses of different genders and age groups. The presence of severe and fatal cases of *P. knowlesi* indicates that additional studies are required to determine whether age, gender or genetic predisposition contributes to disease outcome.

Given that the risk of asymptomatic cases is present in the communities living in region with high *P. knowlesi* cases, including children, there are implications for control and awareness programs in schools and public areas. There can be different comprehension levels in the population which might lead to differing perspectives regarding the infection. Thus, malaria prevention approaches must be customized to target different groups [18, 33]. Children might have an insufficient level of understanding regarding infection, for example, thus acknowledging the community's literacy level and their views of the disease might help to clarify disease issues and improve the understanding of the transmission of the disease. This “bottom-up” approach can help to sustain malaria programs and help to control disease transmission [33].

*Plasmodium knowlesi* exposure is high among households that reside in areas surrounded by forested areas [1]. Public health messages and awareness programmes should be targeted to these communities [14]. Studies should consider the social context that put these communities at risk, despite integrated vector control measures like insecticide-treated nets or long-lasting insecticidal nets, and/or IRS. Considering the views of members of high risk groups can facilitate malaria control in their local settings [34]. Amid concern that current preventive measures might be sub-optimal, community participation coupled with multisectoral strategies can provide more effective malaria control [18]. Deforestation and habitat destruction are causing an increase in the risk of zoonotic malaria transmission occurring [35, 36]. For example, a higher intensity of asymptomatic cases in Sumatra [28], as compared to Aceh, Indonesia [27] was suggested to be due to the deforestation that has occurred in the last few years. Furthermore, *P. knowlesi* cases has been detected for the first time among febrile individuals in Kalimantan, Borneo, and Jambi, Sumatra, Indonesia [37, 38]. As well as residents of forested areas, individuals who travel into the forest may be exposed to zoonotic malaria [38]. Travelers from non-endemic countries should be aware of the risk of zoonotic malaria [39]. Surveillance, especially through the use of sensitive molecular methods is urgently needed in these regions. However, inadequate access to health facilities and malaria treatment may hamper control efforts [40], and these may be exacerbated by misconceptions and community beliefs about supernatural causation of malaria [41].

Poor housing increases malaria risk, and this is also true of zoonotic malaria [38]. However, there may be less risk than with human malarias, as Chua et al*.* suggested that *P. knowlesi* vector mosquitoes bite exclusively outdoors, during the early evening around 1800 to 2200 h—thus suggested the usage of bed nets are not an effective method to control *P. knowlesi* infection [42]. Furthermore, recently, vectors of the *Umbrosus* group were found to carry *Plasmodium* and these species bite earlier, at noon [43]. Therefore, personal anti-mosquito measures such as wearing long sleeves and trousers and the use of anti-mosquito sprays should be encouraged. Communities should be informed of the particular danger of while performing outdoor activities. Qualitative studies in natural settings are required to explore disease exposure, community beliefs, and the spectrum of challenges that put them at risk of mosquito bites [34].

Malaria is a complex disease in which the causative agent, the malaria parasite, is highly genetically polymorphic [44]. This polymorphism leads to a high degree in phenotypic variation that may manifest in the propensity of different strains to cause different levels of pathologies. Antigenic variation, for example, is likely to play a role in maintaining chronic parasitaemia in semi-immune hosts [45], and may also partly explain why asymptomatic malaria may be more common in some areas compared to others [46]. More detailed studies looking at the genetic polymorphism of *P. knowlesi* are required.

As antibodies against *P. knowlesi* are detectable in people living in endemic regions, longitudinal studies may be designed to determine whether the occurrence of these antibodies are associated with disease outcomes, i.e., whether they may be predictive of the development of asymptomatic rather than acute malaria.

One of the limitations of this review is that the study was limited to articles published in English therefore, it could not capture studies published in other languages. The other limitation is that literature search was done from 2000 through 2020, therefore, studies done afterwards are not included. However, to ensure that the systematic review is free from bias and produce a reliable finding, the search strategy was carefully planned, the quality of included articles was checked using quality appraisal tool (STROBE checklist), and reviewed by the authors of this review article.

## Conclusion

*Plasmodium knowlesi* infection is a complex zoonotic disease. Several reports reviewed here reveal parasite carriage in relatively large numbers of people living in endemic areas. This situation warrants further extensive cross-sectional studies to assess the prevalence of asymptomatic *P. knowlesi* parasite carriage, and, perhaps more importantly, other research methodology to better inform understanding of the transmission dynamics of the disease.

It will be extremely difficult to eliminate *P. knowlesi* due to the zoonotic nature of the parasite, but there is an urgent need to improve control measures by including *P. knowlesi* malaria into national malaria control programmes [25].

## Supplementary Information

**Additional file 1. Table S1.** The quality assessment of the included studies using STROBE checklist.

**Additional file 2. Figure S1.** The PRISMA flowchart for systmatic review on asymptomatic *Plasmodium knowlesi* cases.

**Additional file 3. Table S2.** The characteristic of asymptomatic Plasmodium knowlesi cases.

## Data Availability

Not applicable.

## Abbreviations

WHO: World Health Organization
CFR: Case Fatality Rate
WoS: Web of Science
STROBE: Strengthening the Reporting of Observational Studies in Epidemiology
PCR: Polymerase Chain Reaction
LAMP: Loop-mediated Isothermal Amplification
RACD: Rapid Active Case Detection
