# Supplementary material for: A systematic review of asymptomatic Plasmodium knowlesi infection: an emerging challenge involving an emerging infectious disease
Source: Malar J. 2022 Dec 6;21:373. doi: 10.1186/s12936-022-04339-8 (PMC9724390; doi:10.1186/s12936-022-04339-8)
Supplement: Supplementary file 1 — Additional file 1. Table S1. The quality assessment of the included studies using STROBE checklist. [file 12936_2022_4339_MOESM1_ESM.docx]

**Supplementary 1 Table . The quality assessment of the included studies using STROBE checklist**

| STROBE Item | STROBE description | **Eede et al**. (2009)  (11)e | **Fornace et al.** (2016)  (12) | | | **Imwong et al**. (2019)  (14) | | | **Ghinai et al.** (2017)  (17) | | | **Fornace et al**. (2019)  (21) | | | **Fornace et al.** (2018)  (22) | | | **Siner et al.** (2017)  (23) | | | **Marchand et al**. (2011)  (24) | | | **Shimizu et al** (2020)  (25) | | | **Herdiana et al**. (2016)  (26) | | | **Jiram et al**.  (2019)  (27) | | **Lubis et al**  (2017)  (28) | | | **Pongvongsa et al.** (2018)  (29) | | | |  |  |
| --- | --- | --- | --- | --- | --- | --- | --- | --- | --- | --- | --- | --- | --- | --- | --- | --- | --- | --- | --- | --- | --- | --- | --- | --- | --- | --- | --- | --- | --- | --- | --- | --- | --- | --- | --- | --- | --- | --- | --- | --- |
| Item 1 | **Title and abstract** | / | / | | | / | | | / | | | / | | | / | | | / | | | / | | | / | | | / | | | / | | / | | | / | | | |  |  |
| Item 2 | Background and rationale | / | | | / | | | / | | | / | | | / | | | / | | | / | | | / | | | / | | | / | | | | / | | | / | |  | | |
| Item 3 | Objectives or hypothesis | / | | | / | | | / | | | / | | | / | | | / | | | / | | | / | | | / | | | / | | | | / | | | / | |  | | |
| Item 4 | Study design | / | | / | | | / | | | / | | | / | | | / | | | / | | | / | | | / | | | / | | | / | | | / | | | / | |  |  |
| Item 5 | Describe settings, location, relevant dates of recruitment | / | | / | | | / | | | / | | | / | | | / | | | / | | | / | | | / | | | / | | | / | | | / | | | / | |  |  |
| Item 6 | Provides eligibility criteria | / (*P.malariae* cases) | | / | | | NA (malariometric survey) | | | / | | | / | | | NA (comprehensive sampling of all residentsin the study area) | | | NA (longhouses residences with presence of positive *P.knowlesi* in the longhouses) | | | NA (convinience sampling- “as many as possible” | | |  | | |  | | | X | | | NA (convenience sampling) | | | NA (population screening) | |  |  |
| Item 7 | Provides the number of participants/sample size/how the size was calculated | NA ( 95 *P.malariae* cases were screened for *P. knowlesi*( | | / | | | NA (malariometric survey) | | | / | | | / | | | NA | | | NA (longhouses residents) | | | NA (as many as possible) | | | NA (population sscreening) | | |  | | | /NA (Mass blood survey due to outbreak) | | |  | | |  | |  |  |
| Item 8 | Define outcome, exposures, predictors, potential confounders or diagnostic critieria if applicable | / | | / | | | / | | | / | | | / | | | / | | | / | | | / | | | X | | | / | | | / | | |  | | |  | |  |  |
| Item 9 | Sources of data of variables of interest | / | | / | | | / | | | / | | | / | | | / | | | / | | | / | | | / | | | / | | | / | | |  | | |  | |  |  |
| Item 10 | Provides the characteristic of participants at enrollment | / (residents) | | / | | | / (residents) | | | / (residents) | | | / | | | / ( residents) | | | / (residents) | | | / (residents) | | |  | | |  | | | X | | |  | | |  | |  |  |
| Item 11 | Reporting any potential issues on bias | X | | X | | | X | | | / | | | X | | | X | | | X | | | / (gender bias- male) | | | / | | | / | | | X | | |  | | | X | |  |  |
| Item 12 | Plan of analysis | / | | / | | | / | | | / | | | / | | | / | | | / | | | / | | | / | | | / | | | / | | | / | | | / | |  |  |
| Item 13 | Report number of eligible individual, included and completed the study. | NA | | / | | | NA | | | / | | | / | | | NA | | | / | | | NA | | | / | | | / | | | / | | | NA | | |  | |  |  |
| Item 14 | Provides a description of asymptomatic *P. knowlesi* characteristicS | / | | / | | | / | | | / | | | NA (seroprevalence study) | | | / | | | / | | | / | | | / | | | / | | | / | | |  | | |  | |  |  |
| Item 15 | Report numbers of outcome events/summary measures | / | | / | | | / | | | / | | | / | | | / | | | / | | | / | | | / | | | / | | | / | | |  | | |  | |  |  |
| Item 16 | Give unadjusted estimates ( if confounder were adjusted for (OR/RR) or 95% confidence intervals | X | | / | | | X | | | / | | | / | | | / | | | X | | | /x | | | / | | | / | | | X | | | X | | | NA | |  |  |
| Item 17 | Provides a description of asymptomatic *P. knowlesi* infection based on diagnostic method (other analysis) | / | | / | | | / | | | / | | | / | | | / | | | / | | | / | | | / | | | / | | | / | | |  | | |  | |  |  |
| Item 18 | Summarise key result with reference to study objective | / | | / | | | / | | | / | | | / | | | / | | | / | | | / | | | / | | | / | | | / | | | / | | | / | |  |  |
| Item 19 | Discus limitations of the study | X | | / | | | X | | | X | | | / | | | / | | | X | | | X | | | / | | | / | | | X | | | / | | | X | |  |  |
| Item 20 | Overall interpretation of results | / | | / | | | / | | | / | | | / | | | / | | | / | | | / | | | / | | | / | | | / | | | / | | | / | |  |  |
| Item 21 | Recommendation for future research/ generasability (external validity) | / | | / | | | / | | | / | | | / | | | / | | | X | | | / | | | X | | | / | | | / | | |  | | |  | |  |  |
| Item 22 | Funding/conflict of interesr | / | | / | | | / | | | / | | | / | | | / | | | / | | | / | | | / | | | / | | | / | | | / | | | / | |  |  |
|  | Quality of study | S | | S | | | S | | | S | | | S | | | S | | | S | | | S | | | S | | | S | | | S | | | S | | | S | |  |  |

STROBE criteria : Yes (/), No (X), Not applicable (NA)

Quality of study: Sufficient (S), not sufficient (NS)
