# Supplementary figures and images for: A systematic review of asymptomatic Plasmodium knowlesi infection: an emerging challenge involving an emerging infectious disease
Source: Malar J. 2022 Dec 6;21:373. doi: 10.1186/s12936-022-04339-8 (PMC9724390; doi:10.1186/s12936-022-04339-8)

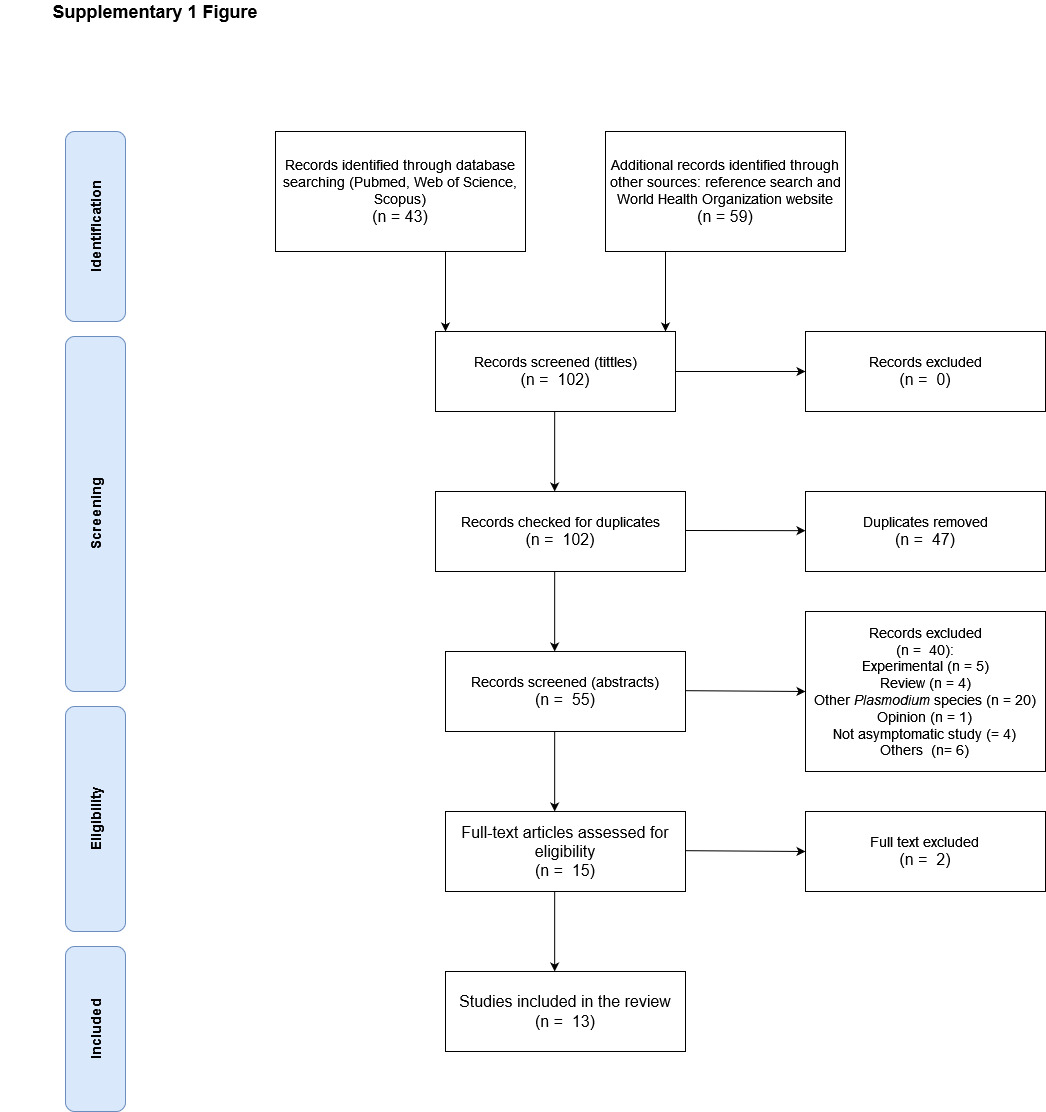

Supplement: Supplementary file 2 — Additional file 2. Figure S1. The PRISMA flowchart for systmatic review on asymptomatic Plasmodium knowlesi cases. [file 12936_2022_4339_MOESM2_ESM.jpg]
