# Supplementary material for: A systematic review of asymptomatic Plasmodium knowlesi infection: an emerging challenge involving an emerging infectious disease
Source: Malar J. 2022 Dec 6;21:373. doi: 10.1186/s12936-022-04339-8 (PMC9724390; doi:10.1186/s12936-022-04339-8)
Supplement: Supplementary file 3 — Additional file 3. Table S2. The characteristic of asymptomatic Plasmodium knowlesi cases. [file 12936_2022_4339_MOESM3_ESM.docx]

**S2 Table: The characteristic of asymptomatic P. knowlesi cases**

| No, citation | Country/ year | Study design | Aims of the study | Diagnostic method | | Primers | | Result | Factors |
| --- | --- | --- | --- | --- | --- | --- | --- | --- | --- |
| Title:  Malaria cross-sectional surveys identified asymptomatic infections of *Plasmodium falciparum, Plasmodium vivax*, and *Plasmodium knowlesi* in Surat Thani, a southern province of Thailand (25) | | | | | | | | | |
| 1.  Shimizu et al. | Thailand/ 2020  " | A population-based,  two **cross-sectional** surveys. One in January (dry season) and the other in May (rainy season) of 2019.  The surveys were conducted in 18 villages in four districts of Surat Thani: Chaiya District (Pak Mak subdistrict), Kirirat Nikhom District (Kapao, Nam Hak and Tha Kanon subdistricts), Vibhavadi District (Takuk Nuea and Takuk Tai subdistricts), and Phanom (Khlong Sok subdistrict) | To determine the prevalence and risk factors of Plasmodium infection in a near-elimination setting in southern Thailand | No BSMP was done, only qPCR followed by nested PCR. [In total, 9418 individuals participated in the study, and 7034 and 8671 blood samples were collected in January and May 2019, respectively.](https://www.sciencedirect.com/topics/medicine-and-dentistry/nested-polymerase-chain-reaction) | | A genus-specific qPCR assay (QMAL), targeting the 18S rRNA | | *P. knowlesi* was detected in one study subject in January 2019 (3%)) and two subjects in May 2019 (4%). Blood samples were positive for *P. knowlesi* by nested PCR method | Being male and staying outdoors at night-time were the only significant risk factors |
| Title: Environmental risk factors and exposure to the zoonotic malaria parasite *Plasmodium knowlesi* across northern Sabah, Malaysia: a population-based cross-sectional survey (21) | | | | | | | | | |
| 2.  Fornace et al. | Sabah, Malaysia/ 2019 | A population-based,  **cross-sectional** survey, environmental-ly stratified across households in the Kudat, Kota Marudu, Pitas, and Ranau districts in northern Sabah, Malaysia | To measure the prevalence of serological exposure to *P. knowlesi* and assess associated risk factors. | Finger prick blood sampling was used to prepare blood smears to detect malaria parasites by microscopy. Whole blood collected into precoated EDTA tubes | | Genus specified 18S ribosomal DNA for nested 1, then antibody response to antibody (SSP-2, SERA3ag2 and AMA-1 | | The PCR method detected three *P.knowlesi* infections (2 mono-infection and one mix infection with *P.vivax*). Seroprevalence of *P knowlesi* was 5·1% in the study population, compromising 10,100 individuals | Age, male sex, contact with macaques, forest use, and raised house construction were positively associated with *P knowlesi* exposure, whereas residing at higher geographical elevations and insecticide use was protective. |
| Title: (14) | | | | | | | | | |
| 3.  Imwong et al. | Cambodia/2019 | Malariometric study, population survey**, cross-sectional** study over 12 months period. | To determine the prevalence and risk factors of *Plasmodium* infection in near elimination setting in Southern Thailand | 14732 samples analyzed using high volume quantitative PCR (qPCR). Parasite detection and genotyping were conducted on blood samples, using qPCR, nested PCR, and were confirmed by nucleotide sequencing | | Primers targeting the gene encoding Plasmodium genus-specific 18s ribosomal RNA (rRNA) | | Eight samples positive for *P. knowlesi* from 14732 samples; Two subjects were females, and six were males detected by PCR; the median age was 35.5 years (range, 23–58 years) | Asymptomatic infections with nonhuman primate malaria parasites *(P.cynomolgi* and *P. knowlesi)* are found in individuals living close to forested areas. |
| Title: Exposure and infection to *Plasmodium knowlesi* in case study communities in Northern Sabah, Malaysia, and Palawan, The Philippines (22) | | | | | | | | | |
| 4.  Fornace et al. | Malaysia (Sabah; Banggi Island and Matunggong and (Limbuak: the Philippines, and September 2014; Palawan/ 2018 | Population survey, **cross-sectional** study | To characterize the community-level patterns of serological exposure to and prevalence of asymptomatic parasitaemia of *P. knowlesi* and other malaria species in three case study communities where *P. knowlesi* transmission has been reported | Thick and thin blood smears and a nested polymerase chain reaction (PCR) method | | Primers targeting the Plasmodium small subunit ribosomal RNA (ssRNA) | | 4 (0.20%) asymptomatic cases found over 2053 samples (2 microscopic positive individuals from Matunggong (Kudat), and PCR positive for *P. knowlesi*, two individuals positive for *P. knowlesi* (PCR) but microscopic negative; a three-year-old girl, and 33 years old woman. | Higher exposure among women and children to asymptomatic *P. knowlesi* infection was reported.  Agricultural work and higher levels of forest cover and clearing around houses is associated with the infection. |
| Title: Malaria risk factor assessment using active and passive surveillance data from Aceh Besar, Indonesia, a low endemic, malaria elimination setting with *Plasmodium knowlesi*, *Plasmodium vivax*, and *Plasmodium falciparum* (26) | | | | | | | | | |
| 5.  Herdiana et al. | Indonesia (Aceh Besar, Sumatra Island)/2016 | Population-based**, cross-sectiona**l study (passive detection using RACD) | To access potential risk factors for any infection, species-specific infection, and secondary case detection | Thin and thick blood smear, and dry blood spots using LAMP and PCR  All pan-LAMP positive samples tested by 18s rDNA nested PCR as well as *P. knowlesi* specific PCR. 10% of LAMP negative (random selected), was performed PCR targeting cytochrome b gene) | | All pan-LAMP positive samples tested by 18s rDNA nested PCR as well as *P. knowles*i specific PCR. 10% of LAMP negative (random selected), was performed PCR targeting cytochrome b gene) | | 1495 individuals enrolled in RACD, 20 (1.34%) positive for *P. knowlesi* (PCR) | Cases are more likely to be male, adult, age 16-45 years compared to <15 years), have visited the forest in the previous month for any reason, and had a workplace near or in the forest and requiring overnight stays. |
| Title: Asymptomatic and submicroscopic carriage of *Plasmodium knowlesi* malaria in household and community members of clinical cases in Sabah, Malaysia (12) | | | | | | | | | |
| 6.  Fornace et al. | Malaysia (Kudat and Kota Marudu)/ 2016 | **Case-control study**; A total of 1147 blood samples were collected from December 2012 through May 2014. | To describe a high level of submicroscop-ic, asymptomatic *P. knowlesi* carriage in an exposed human population | Thick and thin blood smear, nested PCR targeting ssRNA (nested 1), and primers specific for *P. knowlesi* in nested 2. | | Primers targeting the SSU rRNA, cytochrome b nested PCR , and ssu rRNA real time PCR, and chromosome 3 plasmepsin real time PCR (RT-PCR) | | 1147 blood samples were collected, 20 (1.7%) samples were positive for *P. knowlesi*. Prevalence in this population was estimated at 6.9% (95% CI, 5.6%–8.4%). | Presence of a substantial number of asymptomatic *P. knowlesi* mono infections across all age groups. |
| Absence of *Plasmodium inui* and *Plasmodium cynomologi*, but detection of *Plasmodium knowlesi* and *Plasmodium vivax* infections in asymptomatic humans in Betong division of Sarawak, Malaysian Borneo (23) | | | | | | | | | |
| 7.  Siner et al. | Betong Division, Sarawak, Malaysia, 2017 | **Longitudinal study**, 3002 blood sample (dried blood spot) collected from January 2014 – October 2015 | To describe the presence of asymptomatic malaria cases from the longhouse community as most reports are derived from hospitalized patients161 *P. knowlesi* cases were reported 3 years prior to initiation of study | Blood samples on filter paper, nested PCR assay | | Species -specific SSU rRNA | | 7 from 3002 blood samples collected positive for P. knowlesi (6 subjects were afebrile). 1 subject is positive for *P. vivax* | Present asymptomatic *P. knowlesi* and *P. vivax* but not *P. cynomolgi* or *P. inui* within the communities. All cases were adults, and have seen macaques around their farm |
| Contribution of *Plasmodium knowlesi* to Multispecies Human Malaria Infections in North Sumatera, Indonesia (28) | | | | | | | | | |
| 8.  Lubis et al. | Batubara, Langkat, and South Nias regencies, Indonesia, 2019 | **Cross-sectional study**. 3731 participants, among persons attending outpatient clinics in >80 localities, between January -June 2015 across 3 selected regencies in North Sumatera province, Indonesia | 1.To evaluate antimalarial drug efficacy in vivo – an intensive malaria screening in 3 regencies was done  2.To evaluate the sensitivity and specificity of a new *P. knowlesi* specific nested PCR assay, including submicroscopic infection | Dried filter paper blood samples, blood samples for microscopy and PCR (estimated as 0.1 parasites per microL of whole blood) | | 18S rRNA | | 377 participants (11.8%) positive for *P. knowlesi detected by SICavar gene assay, as compare to 76 using rRNA gene. Nearly half of SICAvar gene positive subjects, were also amplicon positive for more than 1 species, where P. vivax being the most common co-infection.* | The amplification of is a specific and sensitive test for the presence of *P. knowlesi* DNA in human. Submicroscopic and asymptomatic multispecies parasitaemia is relatively common in north Sumatera, thus PCR based surveillance is required to support the control and elimination activities |
| Co-infections of *Plasmodium knowlesi, P. falciparum,* and *P. vivax* among Humans and *Anopheles dirus* Mosquitoes, Southern Vietnam (24) | | | | | | | | | |
| 9.  Marchand et.al | Khanh Phu,Vietnam (2011) | 2 study design 1) Active case detection (174 samples)  2) **Cross-sectional** survey (37) from local human population | To describe the mix infection of different *Plasmodium* spp sporozoites in *Anopheles dirus* mosquito and the prevalence of malaria in human | BFMP and blood on filter paper for PCR | | Parasite identification by PCR targeting 18s rRNA  For detection of *P. knowlesi* 18s rRNA, the primer Pmk8 and Pmk9 were used, including circumsporozoi-te protein (CSP) gene | | 4 person has asymptomatic infection from the total of 32 positive *P. knowlesi* cases. 2 of 5 persons were from the Trinh ethnicity, and 2 of 7 were from Kinh ethnicity | *P. knowlesi*–co-infected patients were largely asymptomatic and found among ethnic minority families who commonly spend nights in the forest. They were younger than those infected with other malaria parasite species. |
| Malaria epidemiology in central Myanmar: identification of a multi-species asymptomatic reservoir of infection (17) | | | | | | | | | |
| 10.  Ghinai et al. | Bago region, central Myanmar, 2017 | **Cross-sectional**, household survey during wet season of 2013 using 1,638 household | To estimate the type-specific malaria PCR prevalence and identifying risk factors for seropositivity to malaria | Dried blood spot for PCR | | Parasite identification by targeting the 18s rRNA gene and all positive PCR samples testing using additional method targeting the cytochrome B gene (cytB), designed for this study | | From 41 positive cases by nested PCR, 15 cases were positive during the second stage PCR | There were presence of *P. knowlesi on* discrete demographic risk groups showed opportunities and challenges for malaria control. Responses should target to working-age men as it is capable of detecting sub-clinical infections |
| Human *Plasmodium knowlesi* infections in young children in Central Vietnam (11) | | | | | | | | | |
| 11.  Eede et al. | Vietnam, 2009 | **Cross-sectional study**. 95 randomly selected *P. malariae* samples screened for *P. knowlesi* | To evaluate the effectiveness of long-lasting insecticidal hammocks for controlling forest malaria given around 2004-2006 in Ninh Thuan province, Vietnam | | Nested PCR | | Species-specific SSU rRNA |  | 3 out of 95 *P. malariae* samples are positive for *P. knowlesi*, revealed mix infections. All were asymptomatic. 2 were children. One child still positive one year later |
| Evidence of asymptomatic submicroscopic malaria in low transmission areas in Belaga district, Kapit division, Sarawak, Malaysia (27) | | | | | | | | | |
| 12.  Jiram et al. | Kapit, Sarawak | **Cross-sectional study**. In 2013, 1744 (DBS) collected from 8 longhouses (healthy person).  In 2014, 251 venous blood collected based on 2013 highest number of submicroscopic cases | To investigate the prevalence of asymptomatic submicroscopic malaria in District of Belaga, Sarawak | | BFMP, and dried blood spot | | Nested and nested multiplex PCR | Ss rRNA | 9 of 1744 samples is positive for asymptomatic *P. knowlesi* cases, beside other malaria species. |
| Human infection with *Plasmodium knowlesi* on the Laos-Vietnam border (29) | | | | | | | | | |
| 13. Pongvongsa et al. | Along the Laos-Vietnam border | **Cross-sectional** study among subjects Savannakhet in Laos and Quang Tri in Vietnam between August and October 2010 | To assess the presence of human malaria parasite species and *P. knowlesi* using PCR targeting the 18S small subunit ribosomal RNA gene and circumsporozoite protein gene of the Plasmodium species | | BFMP from finger pricks blood and also applied to filter paper for PCR | | Nest 1, primary amplificati-on using genus-specific primers, rPLU-1/rPLU-5, Nest 2, using species-specific 18S rRNA genes. P. knowlesi detection used two kinds of primer sets, pmk8/pmk9 and Kn1f/Kn3r. The detection of *P. knowlesi* circumsporo-zoite protein (CSP) gene was carried out to confirm the infection | 35/63 positive cases were screened for *P. knowlesi,* including 75 slide-negative samples (family members of microscopy positive patients). Out of 110 samples, 9 positives for *P. knowlesi* who were children (2-10 years old). 7 cases were asymptomatic (based on body temperature) | *M. fascicularis, M. nemestrina, An. dirus* and *An. minimus* are present in the study area. but the asymptomatic *P. knowlesi* cases can be due to genetics of the parasite and the host, and the transmission intensities |
